# Assessing Patient Safety Culture in United States Hospitals

**DOI:** 10.3390/ijerph19042353

**Published:** 2022-02-18

**Authors:** Abdulmajeed Azyabi, Waldemar Karwowski, Peter Hancock, Thomas T. H. Wan, Ahmad Elshennawy

**Affiliations:** 1 Department of Industrial Engineering and Management Systems, University of Central Florida, Orlando, FL 32816, USA; wkar@ucf.edu (W.K.); peter.hancock@ucf.edu (P.H.); ahmad.elshennawy@ucf.edu (A.E.); 2 Department of Health Management and Informatics, University of Central Florida, Orlando, FL 32816, USA; thomas.wan@ucf.edu

**Keywords:** patient safety culture, AHRQ, PLS-SEM

## Abstract

A positive patient safety culture plays a major role in reducing medical errors and increasing productivity among healthcare staff. Furthermore, understanding staff perceptions of patient safety culture and effective patient safety factors is a first step toward enhancing quality of care and patient safety. The objectives of this study were to assess patient safety culture in hospitals in the United States and to investigate the effects of hospital and respondent characteristics on perceived patient safety culture. An analysis of 67,010 respondents in the 2018 Agency for Healthcare Research and Quality (AHRQ) comparative database was conducted with partial least squares structural equation modeling (PLS-SEM). The results revealed that perceptions of patient safety culture had a positive influence on the overall perceptions of patient safety and frequency of event reporting. Moreover, staff position, teaching status, and geographic region were found to have varying influence on the patient safety culture, overall perceptions of patient safety, and frequency of event reporting.

## 1. Introduction

The field of healthcare in the United States (U.S.) has long been considered hazardous because of unhealthful or error-prone environments, high mortality rates, and the unnecessary loss of valuable lives and assets [1]. The inconsistency between highly advanced medical technologies and less developed medical practices leads to disappointment among patients expecting to receive high-quality healthcare, and to frequent vulnerability to medical errors and adverse events (AEs) [1]. AEs not associated with specific diseases include unfavorable outcomes of faulty diagnoses or inappropriate treatments, rather than resulting from medical errors, carelessness, or low-level care [2].

Research has shown that U.S. healthcare institutions lack many of the innovations required to eliminate prevalent risk [2,3,4,5,6]. The Institute of Medicine (IOM) report “*To Err Is Human: Building a Safer Health System*” has brought international attention to the issue of patient safety. By highlighting the amount of harm done, the IOM encourages healthcare institutions to improve the quality of their healthcare practices and thus increase patient safety. Increasing awareness of patient safety creates a culture ensuring that patients encounter fewer risks while receiving healthcare [7]. Globally, attention has shifted to making a culture of patient safety a cornerstone of effective healthcare policy.

Patient safety is defined as “freedom from accidental or preventable injuries produced by medical care” [8], and increased attention has been directed to evaluating safety levels in healthcare organizations [9,10,11]. Increased safety culture awareness has improved healthcare services and led to more favorable outcomes. However, studies and reviews [12,13,14,15,16,17] have found that, globally, large numbers of patients remain vulnerable to avoidable risks and continue to be subjected to below-average levels of healthcare [18], with a rate of AE occurrence between 3% and 17%. For example, in the United Kingdom, a report of the Mid-Staffordshire NHS Foundation Trust has revealed several points of weakness in patient care safety [19]. Ample evidence of wide-ranging errors and failures has been found worldwide [20].

These errors and failures lead to large losses in healthcare assets, costing the U.S. USD 19 billion per year due to hospital overstays, unnecessary time off work, and legal action [21]. Therefore, improving patient safety is an investment in healthcare provision. Policymakers can be assured that the financial gains made by improving patient safety far outweigh the losses [22].

This study sought to highlight the possibilities of establishing institutional healthcare that prioritizes patient safety. In safe and well-established healthcare systems, defects or problems should be detected and addressed in a timely manner, and healthcare services should be continually upgraded and improved to ensure the successful advancement of patient safety [23]. The findings of this study should assist in standardizing medical safety practices to yield more effective and efficient healthcare systems [24,25].

The primary objective of the study was to conduct a patient safety culture (PSC) evaluation by probing the 12 areas assessed by the Hospital Survey of Patient Safety Culture (HSOPSC). The research relied on the development of a model indicating the effects of hospital settings on the respondents. Healthcare workers’ perceptions of PSC and the effects of their awareness of safety culture were thoroughly evaluated.

The research hypotheses were aimed at testing the correlations among potential latent variables, among which PSC was prominent. The latent variable of PSC consists of ten factors: (1) teamwork within the hospital unit, (2) organizational learning and continual improvement, (3) staffing, (4) nonpunitive response to error, (5) communication openness, (6) supervisor/manager expectations and actions promoting safety, (7) feedback and communication regarding errors, (8) management support for patient safety, (9) teamwork across hospital units, and (10) handoffs and transitions. Moreover, the frequency of events reported and overall perceptions of patient safety affect PSC. The study measured the effects of personal and hospital predictors on perceptions of PSC and outcomes in hospital settings. Previous research has indicated that the HSOPSC is considered reliable and valid in the U.S. [5,24,26,27] and internationally [14,23,28,29,30,31,32]. However, no study has examined second-order HSPSC factors. This study was aimed at addressing this gap.

Previous research has indicated that respondent characteristics, such as staff position [33,34], and hospital characteristics, such as teaching status [27,35,36,37] and geographic region [29,30,31,32], significantly influence the perceptions of PSC, frequency of event reporting, and overall perceptions of safety. Because no study has examined the relationships between personal and hospital characteristics, this study was aimed at addressing this gap.

Moreover, PSC has been found to affect the frequency of event reporting and overall perceptions of safety [5,24,26,27,38].

**Figure 1:**
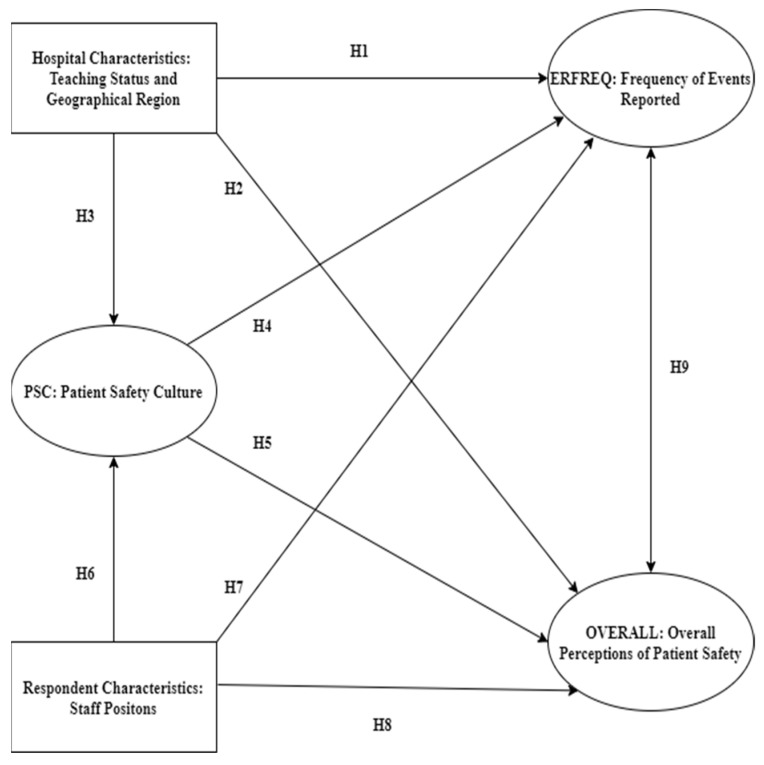
The hypothesized conceptual model.

In view of the above discussion, the relationships among hospital characteristics, respondent characteristics, the frequency of reported error events (ERFREQ), and overall perceptions of patient safety (OVERALL) were explored, on the basis of the following nine hypotheses (Figure 1).

**Hypothesis** **1.** **(H1):** *Hospital characteristics have a significant influence on ERFREQ*.

**Hypothesis** **2.** **(H2):** *Hospital characteristics have a significant influence on OVERALL*.

**Hypothesis** **3.** **(H3):** *Hospital characteristics have a significant influence on PSC*.

**Hypothesis** **4.** **(H4):** *PSC is related to ERFREQ*.

**Hypothesis** **5.** **(H5):** *PSC is related to OVERALL*.

**Hypothesis** **6.** **(H6):** *Respondent characteristics have a significant influence on perceived PSC*.

**Hypothesis** **7.** **(H7):** *Respondent characteristics have a significant influence on ERFREQ*.

**Hypothesis** **8.** **(H8):** *Respondent characteristics have a significant influence on OVERALL*.

**Hypothesis** **9.** **(H9):** *OVERALL and ERFREQ are significantly associated*.

## 2. Materials and Methods

### 2.1. Study Design

This was a retrospective study with a cross-sectional clustered design. The study used a nonprobability convenience sample from the Agency for Healthcare Research and Quality (AHRQ) HSOPSC 2018 comparative database [39,40,41]. The AHRQ began making the HSPSC available to the public in November of 2004 [5]. The AHRQ also established a central repository for comparative databases, maintained by Westat^®^. Westat^®^ is located in Rockville, Maryland, U.S., and it provides research services to agencies of the U.S. Government. HSOPSC data were collected on an annual basis from 2007 to 2018. The call for data collection was extended to every 2 years starting in 2014 [39]. Surveys were administered, and data were cleaned by each hospital in strict accordance with specific AHRQ instructions. Data were then submitted to a central location managed by Westat^®^, where a second level of cleaning was performed [39]. All U.S. hospitals that volunteered to participate in the HSOPSC comparative database were represented in the final dataset.

### 2.2. Study Variables

Three independent variables were extracted from hospital and respondent characteristics: staff position (dummy variable, where medical = 1; nonmedical = 0), teaching status (dummy variable, where teaching = 1; nonteaching = 0), and geographic region. Twelve dependent variables were also extracted, ten of which were dimensions of PSC: (1) teamwork within the hospital unit, (2) organizational learning and continual improvement, (3) staffing, (4) nonpunitive response to error, (5) communication openness, (6) supervisor/manager expectations and actions promoting safety, (7) feedback and communication regarding errors, (8) management support for patient safety, (9) teamwork across hospital units, and (10) handoffs and transitions. The remaining two outcome dimensions were the frequency of reported events and overall perceptions of patient safety.

### 2.3. Participants

Westat^®^, an independent contractor, provided a national repository for the data gathered [39]. To obtain the data, Westat^®^ required the approval of a formal written request. The 2018 U.S. HSOPSC dataset was finalized, officially accepted, and electronically received from Westat^®^ in August of 2020.

The issues related to participation in the survey were handled carefully. Hospital managers chose, at their own discretion, the staff population to participate in the survey, and individual participation was entirely voluntary. Each institution also had complete freedom of choice to participate in the comparative database. All participating hospitals willingly submitted individual-level survey data. Furthermore, hospital managers signed an agreement for data use, consenting to the data being maintained at Westat^®^ and to de-identified data being readily accessible for legal and ethical purposes of healthcare research [42].

Although designated healthcare staff participated in the survey, only de-identified data were used in this research. These de-identified data were supplied solely by Westat^®^ [42].

Data were collected between 2016 and 2018, and the 2018 HSOPSC dataset was found to have an adequate sample size for this study. A total of 630 U.S. hospitals submitted data from 382,834 healthcare staff [39,42]. The available database was statistically adequate to support the complex multi-variable analyses conducted in this study [40].

To meet the study objectives, we stratified the sample on the basis of geographic region, obtaining an adequate representation by region and increasing the generalizability of the findings. The data were divided according to five regions and were then selected with a confidence interval of 99% and a margin of error of 1% for a total sample size of 67,010 respondents, as shown in Table 1.

### 2.4. Statistical Analysis

Descriptive statistics and frequency analyses of the demographic information were performed in IBM SPSS (v.28) [43]; other statistical analyses were conducted in SmartPLS (v.3.3.2) software [44,45,46]. Partial least squares structural equation modeling (PLS-SEM) was used to develop the conceptual model. This tool is considered appropriate for exploratory research and theoretical development, particularly for evaluating numerous variables in a complex model [45]. The Path Weighting Scheme estimation method was used, and significance calculations were achieved through bootstrapping when Smart PLS was used. Sarstedt et al. [45] have recommended that t-statistics be computed with 5000 bootstrap samples; this suggestion was implemented in the current context. Model estimation consisted of a two-step method, with the measurement model first and the structural model second.

In this study, PSC was conceptualized as a reflective hierarchical component model (HCM). The use of an HCM allowed for a less complex and parsimonious path model, particularly given the multi-dimensional construct [44]. PSC is a reflective second-order construct, and its ten dimensions are first-order reflective measurement constructs. A two-stage (or sequential latent score) approach is recommended when the PLS-SEM path model involves a higher order construct [44,45].

## 3. Results

### 3.1. Demographic Variables

The participants were working in teaching or nonteaching hospitals, as shown in Table 2; 56% of participants were from teaching hospitals. The participants were divided into two groups: medical and nonmedical staff (Table 3).

### 3.2. Assessment of Measurement Model

The indicators of the reflective measurement model were highly correlated and interchangeable; therefore, the internal consistency, and the convergent and discriminant validity of the construct required evaluation [44]. Indicator outer loadings between 0.685 and 0.960 are considered acceptable if the average variance extracted (AVE) and composite reliability meet the suggested threshold [45]. As shown in Table 4, the AVE values ranged from 0.536 to 0.905, whereas the composite reliability for all constructs was above 0.8; therefore, all measurement models in this study demonstrated adequate reliability and convergent validity.

Next, the discriminant validity of the reflective constructs was identified with the Fornell–Larcker criterion and the heterotrait–monotrait ratio (HTMT). Table 5 shows that the square root of the AVE for each construct was higher than the correlation values between the latent variables, thus meeting the Fornell–Larcker criterion for discriminant validity. In addition, all HTMT values, as shown in Table 6, were below 0.85, which is the most conservative critical HTMT value [47]. The results showed that the study constructs were conceptually distinct from each other, and discriminant validity for the measurement model was well established.

### 3.3. Assessment of the Reflective Second-Order Construct

This higher order construct was also validated as part of the measurement model assessment, and was assessed for reliability and convergent validity. Furthermore, it was tested for discriminant validity with other lower order constructs, as recommended by Sarstedt et al. [45]. The results established the reliability and validity of the higher order constructs, with a reliability value of >0.70 and a convergent validity AVE > 0.50 (Table 7). For further assessment of reliability and validity, we also assessed the discriminant validity of the higher order constructs with the lower order constructs. The results of the Fornell–Larcker [48] criterion assessment showed that the square root of the AVE of the construct was higher than its correlation with all other constructs (Table 8), whereas the HTMT was also <0.90 (Table 9).

### 3.4. Assessment of the Structural Model

The major evaluation parameters for the structural model included the coefficient of determination (R^2^ value), f^2^ effect sizes, and Q^2^ for the model’s predictive relevance.

The results for the structural model indicated that PSC, hospital characteristics (region and teaching status), and staff position explained 47.6% of the variance in OVERALL, thus indicating the weak predictive power of the model. In addition, OVERALL, PSC, hospital characteristics (region and teaching status), and staff position explained 29.7% of the variance in ERFREQ, thereby indicating that the predictive power was relatively weak. However, hospital characteristics (region and teaching status) and staff position explained 2.9% of the variance in PSC.

The f^2^ values for the hypothesized relationships of PSC with OVERALL and ERFREQ were 0.903 and 0.375, respectively. The results indicated that PSC had a large effect on the R^2^ for OVERALL and ERFREQ. Moreover, the Q^2^ values for ERFREQ and OVERALL were 0.261 and 0.282, respectively, both of which were larger than zero. Therefore, we concluded that the model had good predictive relevance [49,50].

### 3.5. Hypothesis Testing Results

**Figure 2:**
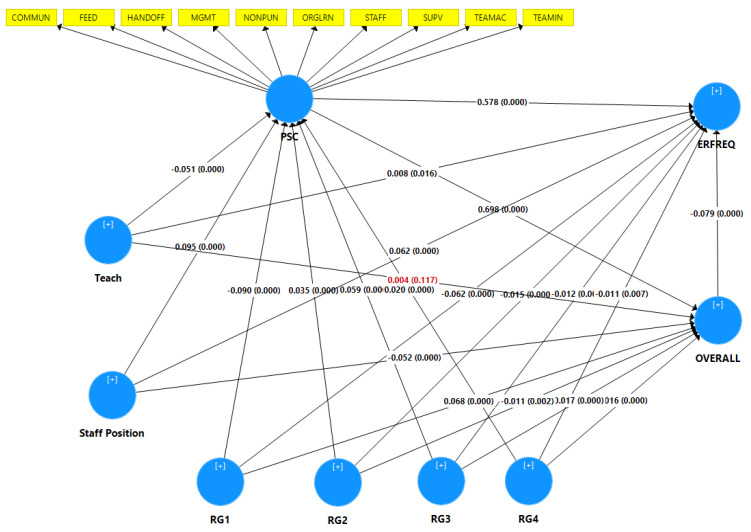
Structural model with path coefficients (β) and *p*-values (*p*). Note: the red values indicate unsupported hypotheses.

We conducted path analysis for all latent predictors to evaluate the correlations among latent variables, given the stated research hypotheses. Moreover, we conducted a bootstrapping test (5000 subsamples were generated) by using PLEase-SEM to measure the validity of path coefficients and to calculate t-test values. After validating the measurement model, we obtained path coefficients (β), t-values (t), and *p*-values (*p*) to determine the appropriateness of the hypotheses. Table 10 shows the estimated path coefficients and t-values between the latent variables. Most of the hypotheses were confirmed by the research findings, with the exception of H2b (Figure 2).

The analyses above yielded the following findings (Table 10):

- H1: Hospital characteristics have a significant influence on ERFREQ.
  A. Regions: The results revealed that staff from all regions had high perception of ERFREQ, with the exception of staff from region one, who had low perception.
  RG1 (β = −0.062, t = 14.031, *p* = 0.000).
  RG2 (β = 0.054, t = 12.575, *p* = 0.000).
  RG3 (β = 0.056, t = 13.237, *p* = 0.000).
  RG4 (β = 0.054, t = 12.694, *p* = 0.000).
  RG5 (β = 0.063, t = 15.188, *p* = 0.000).
  B. Teaching status: The results revealed that staff in teaching hospitals had higher perception of ERFREQ than staff from nonteaching hospitals (β = 0.008, t = 2.312, *p* = 0.021).
- H2: Hospital characteristics have a significant influence on OVERALL.
  A. Regions: The results revealed that staff from region one had a higher perception of OVERALL than staff from other regions.
  RG1 (β = 0.068, t = 18.016, *p* = 0.000).
  RG2 (β = −0.081, t = 20.893, *p* = 0.000).
  RG3 (β = −0.053, t = 14.147, *p* = 0.000).
  RG4 (β = −0.052, t = 13.916, *p* = 0.000).
  RG5 (β = −0.065, t = 17.830, *p* = 0.000).
  B. Teaching status: The results revealed that teaching status did not affect staff perception regarding OVERALL (β = 0.004, t = 1.587, *p* = 0.112).
- H3: Hospital characteristics have a significant influence on PSC.
  A. Regions: The results revealed that staff from all regions had high perception of PSC, with the exception of staff in region one, who had low perception.
  RG1 (β = −0.090, t = 17.249, *p* = 0.000).
  RG2 (β = 0.128, t = 24.994, *p* = 0.000).
  RG3 (β = 0.152, t = 30.491, *p* = 0.000).
  RG4 (β = 0.110, t = 21.485, *p* = 0.000).
  RG5 (β = 0.085, t = 17.457, *p* = 0.000).
  B. Teaching status: The results revealed that nonteaching staff had higher perception of PSC than teaching staff (β = −0.051, t = 12.945, *p* = 0.000).
- H4: PSC is significantly associated with ERFREQ (β = 0.522, t = 147.799, *p* = 0.000).
- H5: PSC is significantly associated with OVERALL (β = 0.698, t = 262.460, *p* = 0.000).
- H6: Respondent characteristics: Medical staff have a significantly higher perception of ERFREQ than nonmedical staff (β = 0.066, t = 19.462, *p* = 0.000).
- H7: Respondent characteristics: Nonmedical staff have a higher perception of OVERALL than medical staff (β = −0.052, t = 17.851, *p* = 0.000).
- H8: Respondent characteristics: Medical staff have a higher perception of PSC than nonmedical staff (β = 0.095, t = 24.090, *p* = 0.000).
- H9: OVERALL and ERFREQ are significantly associated (β = −0.079, t = 14.877, *p* = 0.000).

## 4. Discussion

### 4.1. This Study

The purpose of this study was to investigate the extent of the relationships among the perceptions of PSC, overall perceptions of patient safety, frequency of event reporting, and hospital and respondent characteristics, among medical and administrative staff in U.S. hospitals. The results revealed four aspects underlying these relationships.

First, PSC is a shared value among institutional staff regarding the operation of and interactions between work units and systems, which together produce institutional behavioral norms that promote safety [51]. The results indicated that the perception of PSC has a significant relationship with overall perceptions of patient safety and the frequency of event reporting. The strong correlations indicated that PSC as a higher order construct was valid and reliable for the model, and HOC was used to reduce the number of path model relationships. PSC is associated with procedural efficiency, adequate staffing, managerial support for nurses, and good relationships among staff [52,53,54]. In general, successful hospitals and transparent health systems are those that apply systematic solutions to enhance patient safety [55]. PSC significantly influences safety outcomes, including reporting frequency and overall perceptions of patient safety [51,56,57].

Second, as predicted, hospital characteristics, including region and teaching status, significantly influenced PSC, ERFREQ, and the overall perceptions of participants. The staff in U.S. hospitals had high perception in four of the five regions. Only the staff in hospitals in the Northeast region had low perception of PSC and ERFREQ. However, staff in the Northeast region had higher overall perceptions of patient safety than the staff in other U.S. hospitals. These variations in perception might have occurred because of the diversity of populations, culture, and work experience; therefore, each region should be investigated individually. Wagner [32] has found similar variations in PSC between hospitals in the U.S. and those in the Netherlands and Taiwan, whereas Eiras [29] has found differences in perceptions of PSC among hospitals in northern, central, and southern Portugal. Moreover, staff in teaching hospitals had higher perception of ERFREQ and lower perception of PSC than did nonteaching staff, but teaching status did not influence staff perception regarding overall perceptions of patient safety. These variations can arise from a blaming culture, and educational programs and their availability in health systems. Rather than blaming individuals, a hospital with a positive PSC is open and fair to staff, and learns from its mistakes [52,53]. Güneş [35] has also found no relationship between PSC and hospital type, whereas Ammouri [36] has found that nurses in teaching hospitals are more perceptive of PSC.

Third, participants in this study were divided into medical and nonmedical staff to improve the general understanding of perceptions of PSC. The results revealed that medical staff had higher perceptions of ERFREQ and PSC than nonmedical staff but lower overall perceptions of patient safety than nonmedical staff. These findings implied that hospital administrators/managers differ in their perceptions of the volume and efficacy of error reporting, and their contribution to PSC. These findings are consistent with other research findings suggesting that positive safety settings are associated with increased reporting of medication errors and greater willingness of professionals to advocate for patient safety [27,38,56,58].

Finally, overall perceptions of patient safety showed a significantly negative relationship with the frequency of event reporting. This negative relationship might be due to many reasons, such as the use of self-reported surveys and a blaming culture. Therefore, hospital executives must create cultures in which employees learn from their mistakes, which may increase reporting errors. This finding is consistent with research indicating negative relationships between overall safety culture and patient safety outcomes. For example, hospitals with positive PSC scores have lower rates of in-hospital complications or AEs [27]. In addition, another study has found that a higher safety culture is associated with lower rates of hospital-acquired pressure ulcers [59], and fewer medication or dislodgement errors [60].

### 4.2. Limitations

The present study has several important limitations. The study design included several limitations inherent to the use of secondary data. This was a cross-sectional study including only hospitals that independently administered the survey in the database according to AHRQ’s requirements. The submitting hospitals are not representative of all U.S. hospitals, because they are not a random sample; only approximately 10% of all hospitals chose to participate. Estimates based on this self-selected group might yield biased population estimates, and precise estimates cannot be computed from such a self-selected group. Another limitation was the way in which the surveys were conducted: verification that each hospital followed AHRQ’s data collection procedures could not be obtained, because the investigators overseeing survey distribution were not required to undergo any training. Moreover, another limitation is that the surveys were administered with a variety of methods. Hospitals used paper surveys, Web-based surveys, and a combination thereof. These different modes of administration might potentially have resulted in differences in survey responses; more research is needed to determine whether and how different administration modes affect the results. Finally, this study measured only the subjective overall perception of patient safety, and the frequency of event reporting, both of which were based on only the respondents’ perceptions as an estimate of reporting, rather than actual measurements.

### 4.3. Future Work

We recommend that nonteaching hospitals develop education and training programs for medical and nonmedical staff, collect and statistically analyze error data, and redesign systems to improve PSC, all which can directly decrease the rates of medical errors. This study focused on the perceptions of medical and nonmedical staff. We suggest that future research should focus on nonmedical staff. Hospital administrators and mangers can improve PSC by understanding hospital settings and developing policies and care practices that support programs such as Team Strategies and Tools to Enhance Performance and Patient Safety (TeamSTEPPS^®^). TeamSTEPPS^®^ is a set of evidence-based teamwork tools designed to improve patient outcomes by optimizing interprofessional team functions.

Furthermore, future research should also explore other hospital and respondent characteristics, such as bed size, ownership, and work experience, to establish their effects on perceptions of PSC. This study measured only the subjective overall perception of patient safety and the frequency of event reporting. A future study could include both subjective and objective patient safety indicators. A longitudinal study design including more healthcare professionals and practitioners would be beneficial to increase data reliability.

## 5. Conclusions

This research reveals the different influences of hospital and respondent characteristics on PSC, overall perceptions of patient safety, and ERFREQ. First, the theoretical findings and outcomes were supported by empirical experiments performed with a dimensional approach. Second, this research provides guidance for administrators aiming to improve patient safety. Guidelines include developing partnerships with all stakeholders. The findings and implications should be discussed in the broadest contexts possible. Future research directions should also be highlighted.

## Figures and Tables

**Table 1:** Profile of respondents.

Region	Number of Respondents	Sample Size
1 = Northeast	70,870	13,477
2 = South Atlantic/associated territories	107,584	14,412
3 = East Central	101,984	14,307
4 = West Central	64,091	13,212
5 = West	38,305	11,602
Total	382,834	67,010

Note: Northeast: New England, Mid-Atlantic; East Central: East North Central, East South Central; West Central: West North Central, West South Central; West: Mountain and Pacific.

**Table 2:** Statistics of participants’ hospital status.

Teaching Status	Number of Sample	Percentage
Teaching	37,548	56
Nonteaching	29,462	44
Total	67,101	100

**Table 3:** Statistics of participants’ professional status.

Participants	Number	Percentage
Medical	52,960	79
Nonmedical	14,050	21
Total	67,010	100

Note: Medical: attending/physician/resident/NP or PA; dietician; patient care assistant/hospital aide/care partner; pharmacist; LVN/LPN/registered nurse; therapist. Nonmedical: administration/management; technician (e.g., EKG, laboratory, radiology, unit assistant/clerk/administrative assistant).

**Table 4:** Convergent validity and internal consistency reliability.

Construct	Item	Outer Loading	AVE	CR
Communication openness (COMMUN)	C2	0.899	0.784	0.916
	C4	0.876		
	C6R	0.882		
Feedback and communication about error (FEED)	C1	0.904	0.830	0.936
	C3	0.910		
	C5	0.919		
Staffing (STAFF)	A14R	0.841	0.536	0.821
	A2	0.688		
	A5R	0.685		
	A7R	0.703		
Teamwork across units (TEAMAC)	F10	0.902	0.780	0.934
	F2R	0.866		
	F4	0.901		
	F6R	0.862		
Management support for patient safety (MGMT)	F1	0.890	0.797	0.934
	F8	0.922		
	F9R	0.866		
Nonpunitive response to error (NONPUN)	A12R	0.881	0.748	0.899
	A16R	0.859		
	A8R	0.854		
Organizational learning: continuous improvement (ORGLRN)	A13	0.857	0.668	0.858
	A6	0.825		
	A9	0.768		
Supervisor/manager expectations and actions promoting patient safety (SUPV)	B1	0.902	0.805	0.943
	B2	0.920		
	B3R	0.892		
	B4R	0.873		
Handoffs and transitions (HANDOFF)	F11R	0.880	0.794	0.939
	F3R	0.880		
	F5R	0.906		
	F7R	0.897		
Teamwork within units (TEAMIN)	A1	0.888	0.742	0.920
	A11	0.800		
	A3	0.885		
	A4	0.870		
Overall perceptions of patient safety (OVERALL)	A10R	0.745	0.602	0.858
	A15	0.759		
	A17R	0.814		
	A18	0.785		
Frequency of events reported (ERFREQ)	D1	0.949	0.905	0.966
	D2	0.960		
	D3	0.944		

Note: R = reverse coding item, AVE = average variance extracted, CR = composite reliability.

**Table 5:** Fornell–Larcker criterion and correlations between latent variables.

Construct	COMMUN	ERFREQ	FEED	HANDOFF	MGMT	NONPUN	ORGLRN	OVERALL	STAFF	SUPV	TEAMAC	TEAMIN
COMMUN	**0.886**	-	-	-	-	-	-	-	-	-	-	-
ERFREQ	0.350 **	**0.951**	-	-	-	-	-	-	-	-	-	-
FEED	0.756 **	0.492 **	**0.911**	-	-	-	-	-	-	-	-	-
HANDOFF	0.401 **	0.423 **	0.367 **	**0.891**	-	-	-	-	-	-	-	-
MGMT	0.480 **	0.370 **	0.437 **	0.686 **	**0.893**	-	-	-	-	-	-	-
NONPUN	0.381 **	0.350 **	0.447 **	0.280 **	0.298 **	**0.865**	-	-	-	-	-	-
ORGLRN	0.436 **	0.336 **	0.415 **	0.373 **	0.437 **	0.450 **	**0.817**	-	-	-	-	-
OVERALL	0.455 **	0.317 **	0.403 **	0.396 **	0.462 **	0.517 **	0.661 **	**0.776**	-	-	-	-
STAFF	0.307 **	0.335 **	0.430 **	0.299 **	0.308 **	0.624 **	0.423 **	0.576 **	**0.732**	-	-	-
SUPV	0.530 **	0.285 **	0.477 **	0.350 **	0.445 **	0.392 **	0.474 **	0.483 **	0.352 **	**0.897**	-	-
TEAMAC	0.407 **	0.482 **	0.513 **	0.751 **	0.728 **	0.410 **	0.353 **	0.372 **	0.424 **	0.363 **	**0.883**	-
TEAMIN	0.319 **	0.325 **	0.476 **	0.202 **	0.264 **	0.551 **	0.460 **	0.432 **	0.562 **	0.348 **	0.437 **	**0.862**

Note: The diagonal represents the square root of AVE (bold), and other values indicate the correlations between the variables. ** Correlation is significant at the 0.01 level. COMMUN, communication openness; FEED, feedback and communication regarding error; HANDOFF, hospital handoff and transitions; MGMT, hospital management support; NONPUN, nonpunitive response to error; ORGLRN, organizational learning; SUPV, supervisor/manager expectation and actions promoting safety; TEAMAC, teamwork across hospital unit; TEAMIN, teamwork within unit; STAFF, staffing; OVERALL, overall perception of safety; ERFREQ, frequency of event reporting.

**Table 6:** Heterotrait–monotrait ratio (HTMT).

Construct	COMMUN	ERFREQ	FEED	HANDOFF	MGMT	NONPUN	ORGLRN	OVERALL	STAFF	SUPV	TEAMAC	TEAMIN
COMMUN	-	-	-	-	-	-	-	-	-	-	-	-
ERFREQ	0.385	-	-	-	-	-	-	-	-	-	-	-
FEED	0.856	0.533		-	-	-	-	-	-	-	-	-
HANDOFF	0.451	0.454	0.405	-	-	-	-	-	-	-	-	-
MGMT	0.552	0.407	0.494	0.768	-	-	-	-	-	-	-	-
NONPUN	0.451	0.392	0.515	0.319	0.350	-	-	-	-	-	-	-
ORGLRN	0.539	0.393	0.501	0.446	0.538	0.568	-	-	-	-	-	-
OVERALL	0.553	0.368	0.480	0.469	0.560	0.640	0.859	-	-	-	-	-
STAFF	0.377	0.400	0.526	0.360	0.377	0.793	0.561	0.750	-	-	-	-
SUPV	0.594	0.305	0.524	0.382	0.497	0.448	0.568	0.569	0.421	-	-	-
TEAMAC	0.460	0.520	0.568	0.826	0.818	0.472	0.424	0.441	0.519	0.398	-	-
TEAMIN	0.363	0.353	0.531	0.222	0.298	0.643	0.561	0.515	0.700	0.385	0.486	-

Note: COMMUN, communication openness; FEED, feedback and communication regarding error; HANDOFF, hospital handoff and transitions; MGMT, hospital management support; NONPUN, nonpunitive response to error; ORGLRN, organizational learning; SUPV, supervisor/manager expectation and actions promoting safety; TEAMAC, teamwork across hospital unit; TEAMIN, teamwork within unit; STAFF, staffing; OVERALL, overall perception of safety; ERFREQ, frequency of event reporting.

**Table 7:** Higher order construct reliability and convergent validity.

Construct	AVE	CR
PSC	0.493	0.907

**Table 8:** Fornell–Larcker criterion: higher order discriminant validity.

Construct	ERFREQ	OVERALL	PSC
ERFREQ	0.951	-	-
OVERALL	0.317	0.776	-
PSC	0.534	0.686	0.702

**Table 9:** HTMT: higher order discriminant validity.

Construct	ERFREQ	OVERALL	PSC
ERFREQ	-	-	-
OVERALL	0.368	-	-
PSC	0.583	0.815	-

**Table 10:** Hypothesis testing results.

	Path	F^2^	Path Coefficient (β)	T-Statistic	*p*-Value	Support of Hypothesis by Results
H1a1	RG1 → ERFREQ	0.003	−0.062	14.031	0.000	Supported
H2a1	RG1 → OVERALL	0.005	0.068	18.016	0.000	Supported
H3a1	RG1 → PSC	0.005	−0.090	17.249	0.000	Supported
H1a2	RG2 → ERFREQ	0.003	0.054	12.575	0.000	Supported
H2a2	RG2 → OVERALL	0.008	−0.081	20.893	0.000	Supported
H3a2	RG2 → PSC	0.010	0.128	24.994	0.000	Supported
H1a3	RG3 → ERFREQ	0.003	0.056	13.237	0.000	Supported
H2a3	RG3 → OVERALL	0.003	−0.053	14.147	0.000	Supported
H3a3	RG3 → PSC	0.014	0.152	30.491	0.000	Supported
H1a4	RG4 → ERFREQ	0.002	0.054	12.694	0.000	Supported
H2a4	RG4 → OVERALL	0.003	−0.052	13.916	0.000	Supported
H3a4	RG4 → PSC	0.007	0.110	21.485	0.000	Supported
H1a5	RG5 → ERFREQ	0.004	0.063	15.188	0.000	Supported
H2a5	RG5 → OVERALL	0.005	−0.065	17.830	0.000	Supported
H3a5	RG5 → PSC	0.005	0.085	17.457	0.000	Supported
H1b	Teach → ERFREQ	0.000	0.008	2.312	0.021	Supported
H2b	Teach → OVERALL	0.000	0.004	1.587	0.112	Unsupported
H3b	Teach → PSC	0.003	−0.051	12.945	0.000	Supported
H4	PSC → ERFREQ	0.375	0.522	147.799	0.000	Supported
H5	PSC → OVERALL	0.903	0.698	262.460	0.000	Supported
H6	Staff Position → ERFREQ	0.006	0.066	19.462	0.000	Supported
H7	Staff Position → OVERALL	0.005	−0.052	17.851	0.000	Supported
H8	Staff Position → PSC	0.009	0.095	24.090	0.000	Supported
H9	OVERALL → ERFREQ	0.005	−0.079	14.877	0.000	Supported

## Abbreviations

Adverse events	AEs
Agency for Healthcare Research and Quality	AHRQ
Average variance extracted	AVE
Coefficient of determination	R^2^
Communication openness	COMMUN
Composite reliability	CR
Effect sizes	f^2^
Feedback and communication about error	FEED
Frequency of events reported	ERFREQ
Handoffs and transitions	HANDOFF
Heterotrait–monotrait ratio	HTMT
Hierarchical component model	HCM
Hospital Survey of Patient Safety Culture	HSOPSC
Institute of Medicine	IOM
Management support for patient safety	MGMT
Model’s predictive relevance	Q^2^
Nonpunitive response to error	NONPUN
Organizational learning and continual improvement	ORGLRN
Overall perceptions of patient safety	OVERALL
Partial least squares structural equation modeling	PLS-SEM
Path coefficients	β
Patient safety culture	PSC
*p*-values	*p*
Staffing	STAFF
Supervisor/manager expectations and actions promoting patient safety	SUPV
Team Strategies and Tools to Enhance Performance and Patient Safety	TeamSTEPPS^®^
Teamwork across units	TEAMAC
Teamwork within units	TEAMIN
T-values	t
United States	U.S.
